# Bioinformatics Applications in Life Sciences and Technologies

**DOI:** 10.1155/2016/3603827

**Published:** 2016-05-04

**Authors:** Sílvia A. Sousa, Jorge H. Leitão, Raul C. Martins, João M. Sanches, Jasjit S. Suri, Alejandro Giorgetti

**Affiliations:** ^1^Institute for Bioengineering and Biosciences (IBB), Department of Bioengineering, Instituto Superior Técnico, Universidade de Lisboa, Avenida Rovisco Pais, 1049-001 Lisbon, Portugal; ^2^Instituto Superior Técnico, Universidade de Lisboa, 1049-001 Lisbon, Portugal; ^3^Institute for Systems and Robotics, Instituto Superior Técnico, Universidade de Lisboa, Avenida Rovisco Pais, 1049-001 Lisbon, Portugal; ^4^AtheroPoint, Roseville, CA 95661, USA; ^5^Department of Biotechnology, University of Verona, Strada Le Grazie 15, 37134 Verona, Italy; ^6^Computational Biomedicine, Institute for Advanced Simulation IAS-5 and Institute of Neuroscience and Medicine INM-9, Forschungszentrum Jülich, 52425 Jülich, Germany

Life sciences researchers collect and analyse a high amount of different types of scientific data, including DNA, RNA, and amino acid sequences,* in situ* and microarray gene expression data, protein structures and biological pathways, and biological signals and images of diverse origin. In recent years, a wealth of bioinformatics applications in the fields of basic and applied life sciences has changed the paradigm of both research and exploitation of knowledge. The development of novel and powerful bioinformatics tools dedicated to biological data acquisition, data mining, and analysis empowered both the basic and applied life sciences research. These bioinformatics developments span from tools for genome annotation and function prediction, gene expression analyses, and databases of biological information, to the emerging fields of biomedical applications of research, including the development of new bioinformatics-based devices and predictive applications.

This special issue is composed of five original research papers selected after in-depth peer review. Selected papers describe novel bioinformatics tools and/or databases for fundamental and/or applied research in the broad range of biological and biomedical sciences.

Understanding the genetic control of complex dynamic traits is of fundamental importance to agricultural, evolutionary, and biomedical genetic research. A statistical mapping framework, called functional mapping, has been developed and extensively used to characterize the quantitative trait loci (QTLs) or nucleotides (QTNs) that underlie a complex dynamic trait. However, this tool is not well suited when the curves are complex, especially in the case of nonmonotonic curves. Therefore, to overcome this problem, in their work J. Qi and colleagues propose the earliness index (E-index) to cumulatively measure the earliness degree to which a variable (or dynamic trait) increases or decreases its value. The authors show by both theoretical proofs and simulation studies that E-index is more general than functional mapping and can be applied to any complex dynamic trait, even those with nonmonotonic curves.

RNA-Seq experiments are nowadays extensively used in a wide range of studies, spanning from genome-wide gene expression and regulatory mechanisms underlying basic physiological traits to human pathologies, including cancer. However, RNA-Seq data analyses are complex and require the use of several different tools to manipulate and process the retrieved data. The work presented by F. Russo and collaborators shows recent advancements and novelties introduced in RNASeqGUI, a graphical interface that allows the user to handle and analyse big data sets (collected from RNA-Seq experiments) in a fast, efficient, and reproducible way. The here presented version of RNASeqGUI combines graphical interfaces with tools for reproducible research, such as literate statistical programming, human readable report, parallel executions, caching, and interactive and web-exploitable tables of results.

The Cancer Genome Atlas (TCGA) data portal is a platform containing tumor gene expression data, together with clinical information, enabling researchers to gather information on significant genomic alterations that occur during the development and metastasis of a tumor. To help biomedical researchers to identify gene expression patterns related to breast cancer survival, H. Zhenzhen et al. developed a web-based TCGA data analysis platform called TCGA4U, providing a visualization solution for the analysis of the relationships of genomic changes with the available clinical data. The authors believe that the use of TCGA4U will inspire more biomedical researchers to explore the biological mechanisms of those genes and more precisely explain their role in breast cancer development, paving the way for the discover of more targeted therapies and help more breast cancer patients.

Predicting blood-brain barrier (BBB) permeation is essential for drug design of molecules that act in the central nervous system (CNS). On the other hand, peripherally acting drugs must show limited ability to cross the BBB and therefore be devoid of action in the CNS. However, understanding the process of permeation is complicated, since compounds can cross the BBB by passive diffusion and/or active transport. As an alternative to invasive animal experiments,* in silico* screening methods have been introduced to assist in the development of central nervous system active drugs. In their paper, D. Zhang and colleagues describe the design and implementation of a genetic algorithm to predict the BBB permeation ability of a given molecule, achieving more accurate results than currently available models.

A single nucleotide polymorphism (SNP) is the result of the variation of a single nucleotide at a specific position in the genome. Besides introducing some degree of genetic variation within a population, certain SNPs have been associated with the susceptibility of the individual to specific diseases. The accumulated knowledge resulting from the availability of human genome sequences and the association of specific SNPs with certain diseases prompted the development of the so-called predictive preventive personalized medicine. In their article, P. Ponomarenko and colleagues analysed the effects of SNPs occurring at the promotor region of specific genes on the affinity of the TATA-binding proteins to the promoter region using their web-service SNP_TATA_Comparator. Throughout their work, the authors provide some examples and discuss how to use the bioinformatics application SNP_TATA_Comparator to analyse and extract unannotated SNPs from the database “1000 genomes.” Seventeen novel candidate SNP markers, putatively associated with several diseases, are reported.

The development of bioinformatics tools have changed the paradigm of research in both basic and applied biological sciences, as illustrated by the papers published in this special issue. While these tools enable scientists to gain knowledge of complex biological systems, they also allow envisioning the exploitation of results towards novel developments in biomedical applications, thus contributing to promote both individuals and populations welfare.

*Sílvia A. Sousa* *Jorge H. Leitão* *Raul C. Martins* *João M. Sanches* *Jasjit S. Suri* *Alejandro Giorgetti*

